# The prognostic relevance of HER2-positivity gain in metastatic breast cancer in the ChangeHER trial

**DOI:** 10.1038/s41598-021-92774-z

**Published:** 2021-07-02

**Authors:** Laura Pizzuti, Maddalena Barba, Marco Mazzotta, Eriseld Krasniqi, Marcello Maugeri-Saccà, Teresa Gamucci, Rossana Berardi, Lorenzo Livi, Corrado Ficorella, Clara Natoli, Enrico Cortesi, Daniele Generali, Nicla La Verde, Alessandra Cassano, Emilio Bria, Luca Moscetti, Andrea Michelotti, Vincenzo Adamo, Claudio Zamagni, Giuseppe Tonini, Domenico Sergi, Daniele Marinelli, Giancarlo Paoletti, Silverio Tomao, Andrea Botticelli, Paolo Marchetti, Nicola Tinari, Antonino Grassadonia, Maria Rosaria Valerio, Rosanna Mirabelli, Maria Agnese Fabbri, Nicola D’Ostilio, Enzo Veltri, Domenico Corsi, Ornella Garrone, Ida Paris, Giuseppina Sarobba, Icro Meattini, Mirco Pistelli, Francesco Giotta, Vito Lorusso, Carlo Garufi, Antonio Russo, Marina Cazzaniga, Pietro Del Medico, Mario Roselli, Angela Vaccaro, Letizia Perracchio, Anna di Benedetto, Theodora Daralioti, Isabella Sperduti, Ruggero De Maria, Angelo Di Leo, Giuseppe Sanguineti, Gennaro Ciliberto, Patrizia Vici

**Affiliations:** 1 Division of Medical Oncology 2, IRCCS Regina Elena National Cancer Institute, Via Elio Chianesi 53, 00144 Rome, Italy; 2 Medical Oncology, Sandro Pertini Hospital, Rome, Italy; 3 Oncology Clinic, Ospedali Riuniti Di Ancona, Università Politecnica Delle Marche, Ancona, Italy; 4 Radiation Oncology, Department of Experimental and Clinical Biomedical Sciences, Azienda Ospedaliero-Universitaria Careggi, University of Florence, Florence, Italy; 5 Medical Oncology, Department of Biotechnological and Applied Clinical Sciences, St. Salvatore Hospital, University of L’Aquila, L’Aquila, Italy; 6 Department of Medical, Oral and Biotechnological Sciences, University G. D’Annunzio, Chieti-Pescara, Italy; 7 Medical Oncology Unit B, Policlinico Umberto I, Department of Radiological, Oncological and Pathological Sciences, Sapienza – Università di Roma, Rome, Italy; 8 Breast Cancer Unit, ASST Cremona, Cremona, Italy; 9 Oncology Unit, ASST Fatebenefratelli Sacco Presidio Ospedaliero Fatebenefratelli, Milan, Italy; 10 U.O.C. Medical Oncology, Dipartimento di Medicina e Chirurgia Traslazionale, Università Cattolica del Sacro Cuore, Rome, Italy; 11 Division of Medical Oncology, Department of Oncology and Hematology, University Hospital of Modena, Modena, Italy; 12 UO Medical Oncology I, Transplant and New Technologies Department, S. Chiara Hospital, Oncology, Pisa University Hospital, Pisa, Italy; 13 Medical Oncology Unit, A.O. Papardo, Department Human Pathology, University of Messina, Messina, Italy; 14 Medical Oncology Unit, S.Orsola-Malpighi Hospital, Bologna, Italy; 15 Department of Oncology, University Campus Biomedico of Rome, Rome, Italy; 16 Medical Oncology Unit, Department of Clinical and Molecular Medicine, Sant’Andrea University Hospital, Sapienza Università di Roma, Rome, Italy; 17 Medical Oncology Unit A, Policlinico Umberto I, Department of Radiological, Oncological and Pathological Sciences, Sapienza – Università di Roma, Rome, Italy; 18 Medical Oncology Unit, AOU Policlinico Paolo Giaccone, Palermo, Italy; 19 Department of Ematology and Oncology, Pugliese-Ciaccio Hospital, Catanzaro, Italy; 20 Medical Oncology Unit, Belcolle Hospital, Viterbo, Italy; 21 Medical Oncology Unit, Lanciano-Vasto, Italy; 22 Medical Oncology Unit, Santa Maria Goretti, Latina, Italy; 23 Medical Oncology Unit, San Giovanni Calibita Fatebenefratelli Hospital, Rome, Italy; 24 Medical Oncology Unit, AO S. Croce and Carle Teaching Hospital, Cuneo, Italy; 25 Division of Gynecologic Oncology, Department of Woman and Child Health and Public Health, Fondazione Policlinico Universitario A. Gemelli IRCCS, Rome, Italy; 26 Department of Medical Oncology, ASL Nuoro, Nuoro, Italy; 27 Department of Medical Oncology, IRCCS Giovanni Paolo II Institute, Bari, Italy; 28 Division of Medical Oncology, San Camillo Forlanini Hospital, Roma, Italy; 29 Research Unit Phase I Trials and Oncology Unit, ASST Monza, Monza, Italy; 30 Division of Medical Oncology, Reggio Calabria General Hospital, Reggio Calabria, Italy; 31 Department of Systems Medicine, Medical Oncology, University of Rome Tor Vergata, Rome, Italy; 32 Medical Oncology Unit, ASL Frosinone, Frosinone, Italy; 33 Pathology Department, IRCCS Regina Elena National Cancer Institute, Rome, Italy; 34 Biostatistics Unit, IRCCS Regina Elena National Cancer Institute, Rome, Italy; 35 Fondazione Policlinico Universitario A. Gemelli IRCCS, Rome, Italy; 36 Sandro Pitigliani Medical Oncology Department, Hospital of Prato, Prato, Italy; 37 Department of Radiation Oncology, IRCCS Regina Elena National Cancer Institute, Rome, Italy; 38 Scientific Direction, IRCCS Regina Elena National Cancer Institute, Rome, Italy

**Keywords:** Breast cancer, Cancer therapy

## Abstract

In metastatic breast cancer (mBC), the change of human epidermal growth factor receptor 2 (HER2) status between primary and metastatic lesions is widely recognized, however clinical implications are unknown. Our study address the question if relevant differences exist between subjects who preserve the HER2 status and those who gain the HER2 positivity when relapsed. Data of patients affected by HER2-positive mBC, treated with pertuzumab and/or trastuzumab-emtansine (T-DM1) in a real-world setting at 45 Italian cancer centers were retrospectively collected and analyzed. From 2003 to 2017, 491 HER2‐positive mBC patients were included. Of these, 102 (20.7%) had been initially diagnosed as HER2-negative early BC. Estrogen and/or progesterone receptor were more expressed in patients with HER2-discordance compared to patients with HER2-concordant status (*p* < 0.0001 and *p* = 0.006, respectively). HER2-discordant tumors were characterized also by a lower rate of brain metastases (*p* = 0.01) and a longer disease free interval (*p* < 0.0001). Median overall survival was longer, although not statistically significant, in the subgroup of patients with HER2-discordant cancer with respect to patients with HER2-concordant status (140 vs 78 months, *p* = 0.07). Our findings suggest that patients with HER2-positive mBC with discordant HER2 status in early BC may have different clinical, biological and prognostic behavior compared to HER2-concordant patients.

## Introduction

Breast cancer (BC) heterogeneity is composite in nature, with a wide variety of factors concurring to define several pathological entities, which differ by clinical presentation, pathologic features, therapy administered, and inherent outcomes^1^. Additional sources of breast cancer heterogeneity may raise during the disease course. In BC patients whose disease was initially diagnosed in the early stage and subsequently progressed with metastatic involvement of one single or multiple site/s, the molecular characteristics of metastatic lesions do not necessary mimic those of the disease initially diagnosed.

A well-depicted molecular landscape is crucial for subtype definition, prognostic evaluation and appropriate therapeutic decisions. Accordingly, current guidelines suggest repeating the immunohistochemical (IHC) assessment in patients with metastatic spread and at least one secondary lesion amenable to biopsy^2^. Discordance in human epidermal growth factor receptor 2 (HER2) status between the tumor and metastatic lesions is widely acknowledged, and not yet completely unraveled in their biologic meaning and prognostic relevance^3–11^. The overexpression of HER2 or amplification of the related gene is extensively recognized as a feature associated with more aggressive biological behavior^12,13^. However, the extent to which changes in HER2 status may affect patients’ prognosis is still a matter of debate^14^.

We herein propose an observational study of HER2-positive metastatic breast cancer (mBC) patients treated with the anti-HER2 targeted agents pertuzumab and/or trastuzumab emtansine (T-DM1). Our research question is whether relevant differences exist in long-term outcomes of patients with concordant HER2 status between the primary tumor and its secondary lesion/s compared to patients whose disease revealed HER2-positivity gain at the IHC assessment of metastatic lesions. In our historical cohorts, we also sought to identify factors associated with HER2-positivity gain at the IHC reassessment, for which an impact on prognosis may be foreseen.

## Results

From 2003 to 2017, 491 HER2‐positive mBC patients were retrospectively identified at the participating centres. Overall, 102 (20.7%) patients had been initially diagnosed with a HER2-negative early BC, while 389 (79.2%) patients were HER2-positive also at the time of initial diagnosis.

The clinical-pathological features of the overall cohort (N = 491) by HER2 status in the early and advanced settings are reported in Table 1A,B, respectively. Briefly, when analyzing the IHC characteristics, we observed that ER and/or PgR were more commonly expressed in patients with HER2 status discordance between the early and metastatic disease compared to patients with concordant HER2 status (*p* < 0.0001 and *p* = 0.006, for ER and PgR, respectively). Patients with HER2 discordant tumors showed more frequently a triple positive (TP) subtype at metastatic diagnosis, i.e., expressed more often both ER and PgR, compared with their counterpart (53% *vs* 40.6%, *p* < 0.0001). Moreover, we found a significantly higher rate of brain metastases in patients with HER2-concordant tumors compared to HER2-discordant cancers (26.7% *vs* 14.7%, respectively; *p* = 0.01). There was evidence of more common visceral metastases in patients with HER2-discordance than in patients with concordant HER2-status (75.5% *vs* 65.3%, *p* = 0.05). Conversely, no differences were highlighted in terms of bone metastasis distribution (*p* = 0.38). Statistically significant differences emerged when analyzing the disease-free interval (DFI), with longer DFI in patients with HER2-discordant tumors (*p* < 0.0001). Treatments administered to the whole study population in first-and second-line are reported in supplementary Table 1 and supplementary Table 2 Median follow-up for the entire study population was 36 months (range, 3–256).

**Table 1:** Clinic-pathological characteristics of the study participants according to HER2 status at initial diagnosis (1A) and when metastatic (1B) (N:491).

Characteristics	HER2-negative, 102 pts [N (%)]	HER2-positive, 389 pts [N (%)]	*p*-value
**Section A. Characteristic of BC at initial diagnosis**			
**Age**			
* ≤ *65	96 (94.1%)	354 (91%)	0.37
* > *65	6 (5.9%)	35 (9%)	
**Histological subtype**			
Ductal	82 (80.4%)	340 (87.4%)	0.11
Lobular	7 (6.8%)	18 (4.6%)	
Other	13 (12.8%)	31 (8.0%)	
**Estrogen Receptor**			
Positive	82 (80.4%)	232 (59.6%)	< 0.0001
Negative	20 (19.6%)	157 (40.4%)	
**Progesterone Receptor**			
Positive	58 (56.9%)	162 (41.6%)	0.006
Negative	44 (43.1%)	227 (58.4%)	
**Neo-/adjuvant treatment**			
Yes	91 (89.2%)	350 (90%)	0.82
No	11 (10.8%)	39 (10%)	
**Neo-/adjuvant trastuzumab**			
Yes	0	259 (66.6%)	< 0.001
No	102 (100%)	130 (33.4%)	
**Section B. Characteristics of BC when metastatic**			
**IHC Subtype**			
TP	54 (53.0%)	158 (40.6%)	
ER or PgR positive	28 (27.4%)	74 (19.0%)	< 0.0001
ER and PgR negative	20 (19.6%)	157 (40.4%)	
**Visceral metastases**			
Yes	77 (75.5%)	254 (65.3%)	0.05
No	25 (24.5%)	135 (34.7%)	
**Bone metastases only**			
Yes	4 (3.9%)	24 (6.2%)	0.38
No	98 (96.1%)	365 (93.8%)	
**Brain metastases**			
Yes	15 (14.7%)	104 (26.7%)	0.01
No	87 (85.3%)	285 (73.3%)	
**Number of metastatic sites**			
1	72 (70.7%)	275 (70.7%)	
2	17 (16.6%)	72 (18.5%)	0.81
> 2	13 (12.7%)	42 (10.8%)	
**Disease Free Interval**			
< 3 years	24 (23.5%)	182 (46.7%)	< 0.0001
≥ 3 years	75 (73.5%)	193 (49.6%)	

N, Number; BC, breast cancer; IHC, Immunohistochemical; TP, triple positive; ER, estrogen receptor; PgR, progesterone receptor.

Overall, in the 491 patients who contributed data to our analysis, median PFS at first‐line treatment was 10 months (95% CI, 3–96), with no significant differences by baseline HER2 status (11 months in both subpopulations, *p* = 0.24). Among the evaluable patients, median OS (mOS) was 91 months (95% CI, 71–110). When analyzing patients’ data according to HER2-status at baseline, we found some evidence of advantage in patients with discordant HER2-status, with a mOS of 140 months (95% CI, 61–220) with respect to patients with concordant HER2-status, whose mOS was 78 months (95% CI, 59–97) (*p* = 0.07).

We further addressed the outcomes of patients who received the anti-HER2 targeted agents according to the currently recommended treatment sequence, namely, pertuzumab-based therapy in first line and T-DM1 in second line. In this subset (N:117), the 3-year cumulative survival rate of patients (N:34) with tumors exhibiting a discordant HER2-status was 73%, which dropped to 49% in patients (N:83) with HER2-positive tumors at initial diagnosis.

Results from uni- and multivariate analyses are shown in Table 2. Univariate models confirmed longer mOS in patients who were HER2-negative at baseline even if at a not fully significant extent (HR 1.51; 95%CI 0.96–2.37; *p* = 0.072). Factors impacting on mOS negatively were the presence of brain metastases (HR 2.09; 95%CI 1.51–2.88; *p* < 0.001), and shorter disease free survival (DFS) (HR 1.69; 95%CI 1.23–2.32; *p* = 0.001), which were both confirmed in multivariate analysis (HR 1.98; 95%CI 1.43–2.74; *p* < 0.001 and HR 1.68; 95%CI 1.22–2.32; *p* = 0.001, respectively).

**Table 2:** Uni- and multivariate Cox regression models for OS.

		Univariate Cox regression model		Multivariate Cox regression model^a^	
		HR (95%CI)	*p*-value	HR (95%CI)	*p*-value
Age	> 65 years vs ≤ 65 years	1.03 (0.56–1.91)	0.91		
IHC subtype	TP vs ER or PgR + vs other	–	0.92		
	ER or PgR + vs TP	0.99 (0.65–1.51)	0.97		
	Other vs TP	1.07 (0.75–1.51)	0.72		
ER	Negative vs Positive	1.07 (0.77–1.48)	0.69	–	–
PgR	Negative vs Positive	1.04 (0.76–1.42)	0.80	–	–
Visceral metastases	Yes vs No	1.11 (0.79–1.58)	0.54	–	–
DFS	< 3 years vs ≥ 3 years	1.69 (1.23–2.32)	0.001	1.69 (1.23–2.32)	0.001
HER2 + at baseline	Yes vs No	1.51 (0.96–2.38)	0.072	–	–
Brain metastases	Yes vs No	2.09 (1.51–2.89)	< 0.0001	1.98 (1.43–2.75)	< 0.0001

HR, hazard ratio; IHC, Immunohistochemical; TP, triple positive; ER, estrogen receptor; PgR, progesterone receptor; DFS, disease free survival.

^a^Adjusted for the variables significant at the univariate analysis.

Since DFI showed a significant impact on OS in univariate analysis and was confirmed in multivariate models, we focused on the impact of adjuvant trastuzumab on the outcome of interest. We thus newly evaluated overall survival (OS) excluding patients who had received adjuvant trastuzumab. When doing so, as shown in Supplementary Fig. 1, no significant differences emerged between originally HER2-positive patients (N:130) and originally HER2-negative patients (N:101) in terms of 5-year and 10-year OS (*p* = 0.43). The results observed encouraged further analysis. In our case-series, 389 patients were HER2-positive at initial diagnosis. Among them, 259 had received adjuvant trastuzumab, while 130 had not. When compared by administration of adjuvant trastuzumab, these two groups differed significantly in terms of OS. Median OS estimates were 110 months (95CI% 72–148) and 60 months (95% CI 50–70), for patients not having received and treated with adjuvant trastuzumab, respectively (*p* < 0.0001). Apparently, our data elicited a survival advantage in HER2-positive patients who had not received adjuvant trastuzumab compared to their counterpart. On this basis, we compared the 2 groups, i.e., HER2-positive patients not having received adjuvant trastuzumab, and HER-2 positive patients having received adjuvant trastuzumab by relevant clinical-pathologic features. In patients who received adjuvant trastuzumab, we observed more commonly brain metastases (30.6% vs 19.2%, *p* = 0.02), and less frequent hormone receptor expression (ER: 52.1% vs 74.6%, *p* < 0.0001 and PgR: 35.5% vs 53.8%, *p* = 0.001). Consistently, HER2 enriched cases were more common among women treated with adjuvant trastuzumab, while luminal cancers were less represented (*p* < 0.0001). Finally, in patients treated with adjuvant trastuzumab, disease free survival was more commonly shorter than 3 years (59.6% vs 26.4%, *p* < 0.0001).

## Discussion

We analyzed data from a historical cohort of 491 HER2‐positive mBC patients treated with pertuzumab‐based regimens and/or T‐DM1 according to standard clinical practice. We specifically focused on whether HER2-positivity gain in mBC patients was associated with significantly different long-term outcomes as compared to those of patients with concordant HER2-status between the primary tumor and its associated secondary lesions. Overall, HER2-positivity gain was verified in a not negligible percentage of patients from our case series, i.e., 20.7%. In these patients, hormonal receptors were more frequently expressed at the IHC assessment, either singularly or contemporarily, compared to their counterpart. Further differences emerged when comparing these two groups by DFI and pattern of metastatic spread, with a more favorable profile in patients with an initially HER2-negative disease. We found some evidence of longer mOS in patients with HER2-positivity gain in the metastatic setting, although at a not fully statistically significant extent (*p* = 0.07). This evidence was confirmed and statistically reinforced in patients who received pertuzumab-based therapy in first line and T-DM1 in second line, with a 3-year survival rate of 73% in patients with discordant HER2-status and 49% in patients with concordant HER2-status. Brain metastases and shorter DFS were both associated with less favorable mOS in uni- and multivariate models.

Taken together, our findings suggest distinct clinical-biological characteristics and outcomes of HER2-positive mBC patients first diagnosed with a HER2-negative early disease compared to patients with an initial HER2-positive early disease, with more favorable mOS in the former group. Prior studies on HER2 status discrepancies between the primary tumor and associated secondary lesions have mainly addressed the frequency of this phenomenon, often neglecting aspects related to its prognostic relevance and factors influencing its occurrence. In a systematic review and meta-analysis including 39 studies, the discordance rate for HER2 was assessed in 2,440 patients. The inherent random effects pooled positive to negative conversion percentage was 21.3% (95% CI = 14.3% to 30.5%), while the negative to positive conversion percentage was 9.5% (95% CI = 7.4% to 12.1%)^15^. The estimated rate thus appears substantially lower compared to ours, i.e., 20.7%. In our study, the denominator exclusively included mHER2-positive breast cancer cases treated with pertuzumab and/or T-DM1 in clinical practice. More generally, prior works have shown widely varying HER2 discordance rates, which may raise up to 24%, as reported by Niikura and colleagues^16^. Remarkable variations are more often described in bone metastases, at least partially due to the technical interference related to decalcification, which may reflect on the reliability of the IHC assessment. However, most of the authors convene on that the conversion to positive HER2-status is, on average, lower than the negative conversion. This reinforces the hypothesis that tumor heterogeneity may be a crucial node of this phenomenon^16–18^. Tumor heterogeneity may be due to tumor biological drift, selective pressures of therapy leading to clonal selection and development of novel tumor cell clones, or presence of small subclones routinely undetected within the primary tumor^19^. The occurrence of mechanisms of resistance could partially explain this phenomenon, as the result of temporal tumor heterogeneity that fuels the development of metastatic clones which do not express HER2. Conversely, next-generation sequencing studies strengthened the hypothesis that variation in HER2 status may reflect a clonal genome evolution. Whether HER2 discordance reflects the mechanism of treatment resistance or heterogeneity of expression within the primary site is still unclear.

As anticipated, the evidence on the prognostic relevance of HER2 discrepancies between primary cancer and secondary sites is poor and not always consistent. Edgerton and co-authors examined differences in HER2-status between recurrent or metastatic disease compared with the primary tumor in 113 breast cancer patients. The longest PFS and OS were observed in patients whose primary tumor and metastases were both HER2-negative, while the most unfavorable outcomes were reported in patients whose metastatic disease showed a positive to negative conversion^20^. Chang and colleagues addressed the topic of discrepancies in hormonal receptors and HER2 and response to trastuzumab in fifty-six patients The HER2 status resulted discordant between primary and secondary lesions in seven patients (12.5%). Among them, five patients developed a negative to positive conversion and received trastuzumab-based chemotherapy. Overall, discordant HER2 status was associated with less favorable outcomes^21^. Lower and colleagues compared original primary tumours with subsequent metastatic lesions from 382 patients by HER2 status. Survival differences were revealed, with the most favorable outcomes been observed in patients with HER2-negative primary tumor and subsequent HER2-positive metastatic lesions^22^. The quite limited number of study participants in the studies from Edgerton and Chang as compared to the studies from Lower and our group may at least partly explain inconsistency in results. In addition, technical issues related to HER2 status assessment should be considered at least for the oldest study^20^. When coming to treatment administered, the use of the anti-HER2 targeted agent pertuzumab and T-DM1 makes our study barely comparable to prior works, as discussed later. The significant advantage in mOS observed when comparing discordant and concordant cases exclusively from the subgroup treated with pertuzumab and T-DM1 according to the recommended treatment sequence seems to further orient toward a relevant role of targeted therapy in affecting the outcomes of interest. In our historical cohort, mBC patients experimenting HER2 gain expressed hormonal receptors more commonly than their counterpart. Van Rooijen et al. assessed concordance of HER2 status in a population based sample of 174 mBC patients, who resulted HER2-positive at local assessment and were treated with trastuzumab. Consistently with our findings, these authors found a higher rate of hormone receptor expression in HER2-discordant patients compared to HER2-concordant patients. This evidence was deemed biologically plausible since hormone receptor positivity is generally higher in truly HER2-negative mBC compared to HER2 positive mBC^23,24^. In our historical series, HER2-discordant patients also showed more favorable profiles in terms of DFI and metastatic spread as compared to patients with concordant HER2-status. This may reflect a clonal evolution of the disease, which could evolve by developing a more aggressive behavior. The selective pressure exerted by prior therapy/ies deserves further note. In a recent study of 270 mBC patients who underwent re-biopsy at disease progression, the evidence observed suggested that adjuvant endocrine treatment positively correlated with PR discordance, while ER discordance was associated with chemotherapy. Persistent ER negative status was associated with unfavorable survival outcomes. The authors found no evidence of an impact of HER2 conversion. This may be partly at least explained by the fact that, in most of these patients, HER2 conversion did not affect therapeutic decisions in terms of targeted agents administration^25^.

Our study suffers from some limitations. The retrospective design makes it prone to confounding and bias. Some among the potential sources of bias deserve further discussion. First, there is an undeniable attitude to exclusively characterize those metastases amenable to biopsy, namely, secondary lesions for which biopsy sampling does not contrast at any extent with patients’ safety. In addition, some patient- and disease-related characteristics may more definitely invite re-biopsies. It is plausible and common in clinical practice that patients with better PS, longer DFI, and lower disease burden, e.g., single metastasis or possible second cancer, are more frequently re-biopsied compared to their counterparts. When globally considering the aforementioned issues, and in specific referral to the coordinating centre, i.e., the IRCCS Regina Elena National Cancer Institute, the average percentage of metastatic breast cancer patients who undergo re-biopsy is set at about 60%. A further source of bias emerged when addressing the impact of adjuvant trastuzumab on OS in HER2-positive metastatic breast cancer patients who had been first diagnosed with a HER2-postive disease. Unexpectedly, survival outcomes of patients treated with trastuzumab were significantly worse than those of the counterpart. A first plausible explanation may be provided by the less favorable characteristics of this subgroup in terms of relevant clinical-pathological features. We may also add that this subgroup of patients, differently from its counterpart, was not naïve to anti-HER2 agents, given the prior exposure to adjuvant trastuzumab and was less commonly amenable to hormone therapy, due to the higher representation on HER2-enriched breast cancers. It may be worth mentioning that about 60% of initially HER2-postive patients who did not received adjuvant trastuzumab had been diagnosed prior to the year 2005, that is, prior to the AIFA approval relatively to the use of adjuvant trastuzumab in HER2-positive breast cancer. In the attempt to mitigate the impact of the previously mentioned sources of bias, several strategies were adopted. Data retrieving from clinical records was exclusively performed by ad hoc trained research personnel and based on the use of pilot-tested forms. In addition, techniques of stratification and adjustment at the time of statistical analysis allowed to control for confounding. Our results are also clouded by the absence of standardization of HER2 evaluation, performed by different pathologists at several centers. In addition, particularly in light of the quite long DFI recorded for some of our patients, differences in methods of HER2 status assessment overtime must be considered^12,13^. However, routine quality controls are in place at the participating centres. This increases our confidence in data quality. Furthermore, treatments received in both the adjuvant and metastatic settings were various. Treatment decisions are highly conditioned by a number of factors, partly defined at an individual patient level, e.g., age, co-morbidities, and also influenced by the physician experience and choices. All of these factors are amply contemplated in the real world setting and overall acceptable as a potential source of variability. A further issue deserves discussion. In our study, all participants were mBC patients treated with pertuzumab and/or T-DM1. For each of them, we retrospectively verified the HER2 status prior to pertuzumab or T-DM1 treatment. In 102 of them, those with a discordant HER2 status, re-biopsy had been performed, HER2 status newly assessed and treatment assigned accordingly. For the remaining 389 patients, who resulted HER2 positive at an earlier assessment, re-biopsy was not performed. In this latter group, anti-HER2 treatment with pertuzumab and/or T-DM1 was driven by the results of the earlier assessment. In clinical practice, targets evaluation is routinely performed at the time of initial diagnosis, using more commonly tissue samples from the primary tumor. In solid tumor management, performing multiple biopsies over a patient life-span represents, at least partly, a still questionable approach. Re-biopsy is an effective tool for assessing the histology and molecular landscape of secondary lesions and eventually highlighting HER2, or, more generally, molecular discrepancies between the disease at its onset and at the time of progression. Thus, re-biopsy holds the potentials to be informative concerning the disease nature, prognosis, persistence and/or gain of therapeutic targets. However, the feasibility of this procedure is highly dependent on the presence of easily accessible metastatic lesions, to be evaluated in the full respect of patients’ safety. As previously mentioned, the positive to negative conversion of previously identified targets is more common than its opposite. However, in the case of targets verified at time of diagnosis, particularly in the only ones, a positive to negative conversion at disease progression does not necessarily exclude continuing to exploit the initially identified targets for therapeutic purposes. Conversely, in the case of a negative to positive conversion, namely, new targets acquisition, the extent to which the switch to therapeutic options implying new target exploitation may translate into significant outcome amelioration is still to be defined^18^.

**Figure 1:**
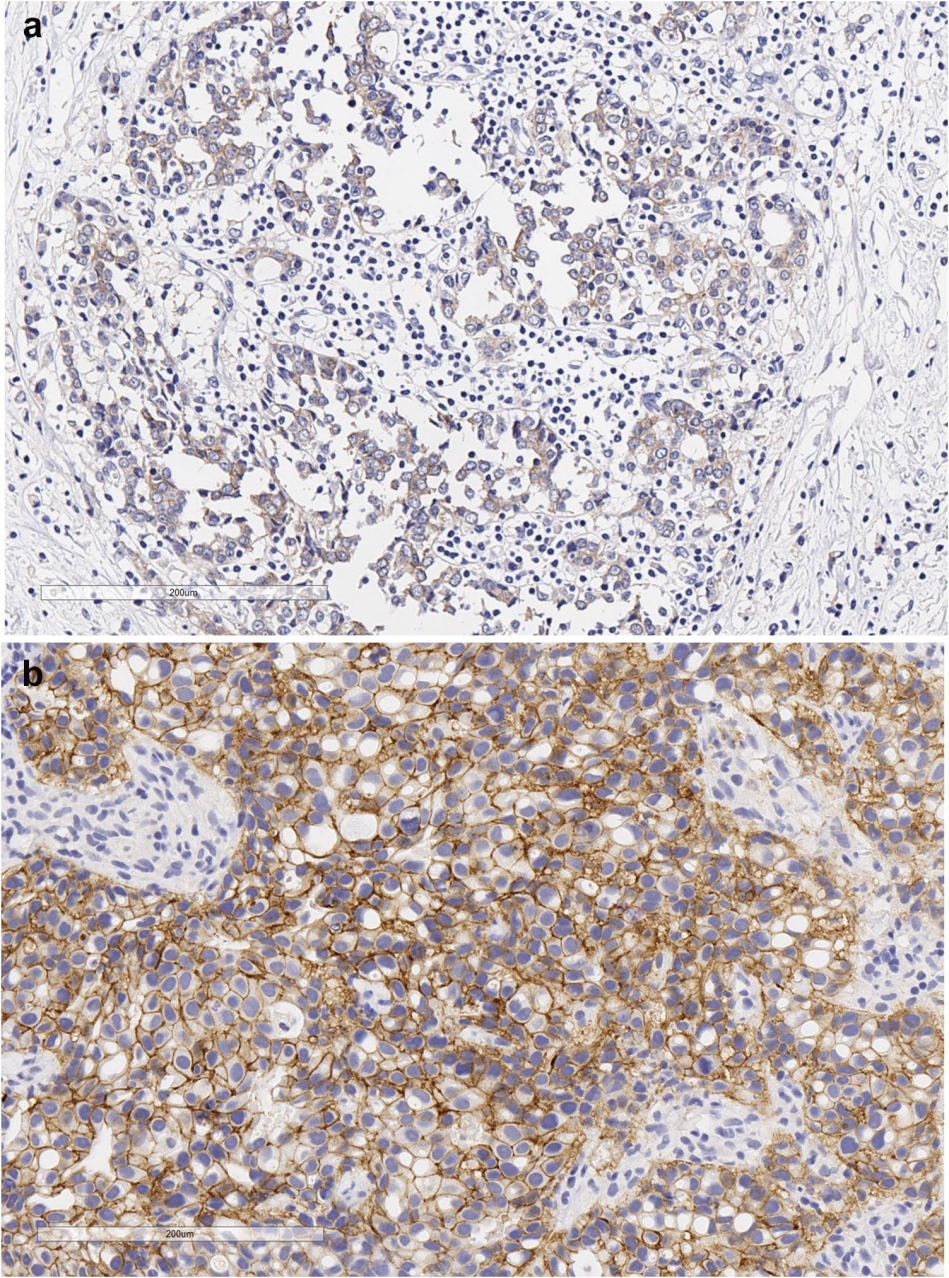
Histopathology features of a patient who switch the HER2 status from negative early breast cancer to positive in metastatic setting. In (**a**), at breast cancer diagnosis, HER2-assessment resulted as “Membrane staining that is incomplete and barely perceptible and within ≤ 10% of tumor cells. IHC: 0 negative”. At relapse (**b**), soft tissues from the left parasternal region were biopsied. HER2 expression resulted moderate, completed in > 10% of tumor cells. IHC: 2 + . The FISH resulted into gene amplification.

**Figure 2:**
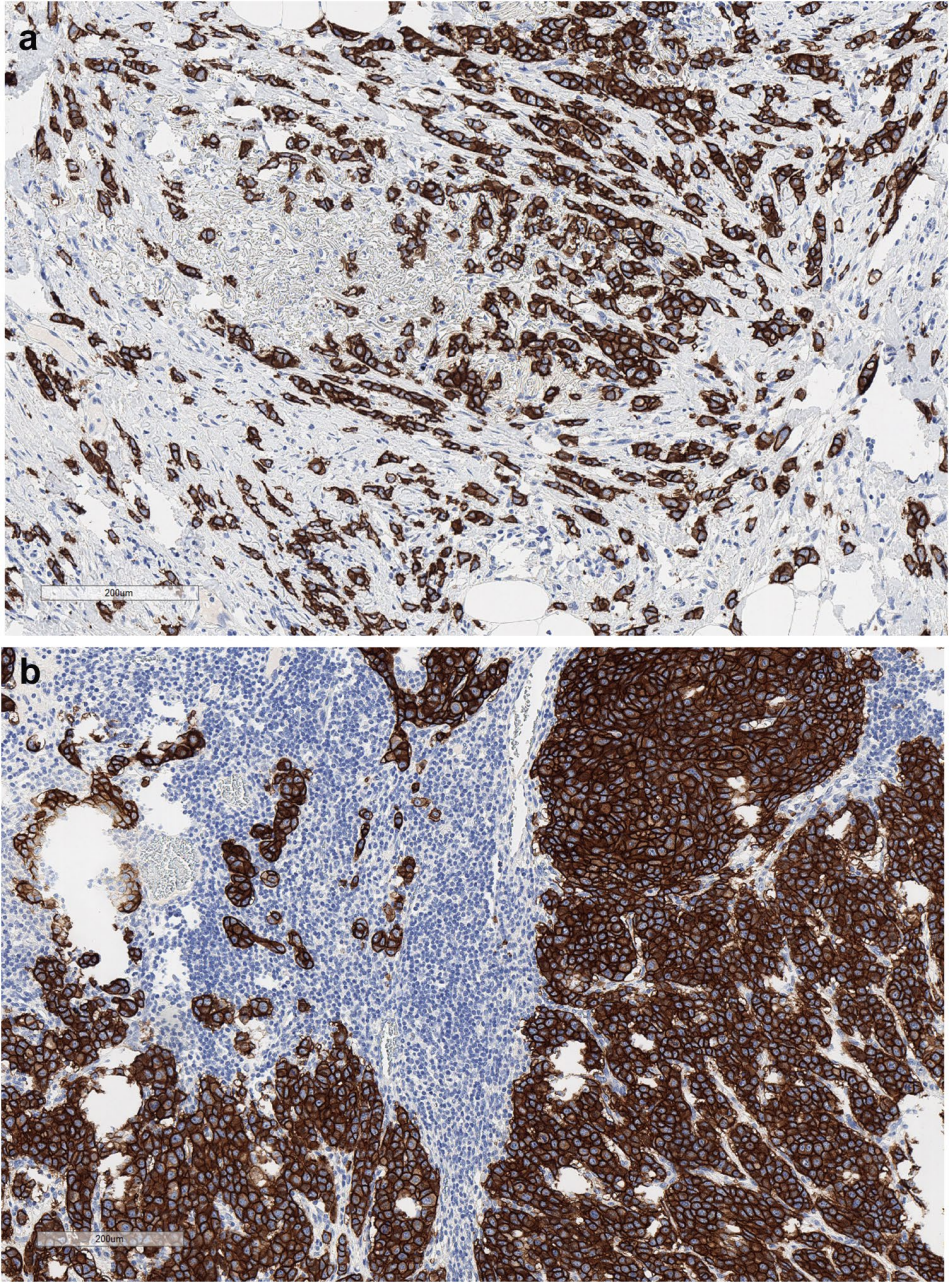
Histopathology example of a patient who maintained the HER2 status both in early and metastatic biopsy. At breast cancer diagnosis (**a**) the pathologists’ report called for “Membrane staining that is complete, intense and in ˃ 10% of tumor cells. IHC: 3 + .” At relapse (**b**), a cutaneous lesion in the ipsilateral breast was biopsied and the pathologists’ report exactly reproduced the prior one.

Our study also has considerable strengths. The collaborative efforts of 45 Italian centres allowed for the inclusion of 491 HER2-positive, mBC participants. To our knowledge, this makes our study the largest conducted thus far on the topic of interest. The use of the new generation anti-HER2 targeted agents pertuzumab and/or T-DM1 in our historical cohorts makes our study unique, since prior studies on HER2 status discordance between the primary tumour and secondary lesions have included patients treated with trastuzumab. In addition, far beyond the rate of phenomenon of negative to positive HER2 conversion, we focus on its prognostic relevance and show evidence related to factors which seem to impact mOS both in uni- and multivariate analyses. The quantitative evaluation of HER2 status would have greatly enriched the present study. We actually refrained from covering such relevant tasks because of some intrinsic study limitations, mostly related to its multicentric nature and observational, retrospective design. Indeed, particularly relevant questions remain concerning the impact of the metastatic site not only on HER2 qualitative assessment, i.e., change/stability of HER2 status at the time of disease relapse/progression compared to the HER2 status at initial diagnosis, but also on some potentially relevant quantitative aspects. Due to the previously recalled limitations, we were exclusively able to provide images in support of two cases, i.e., a patient initially diagnosed with a HER2-negative tumor, who tested positive for HER2 when assessed at the time of relapse (Fig. 1a,b), and a case of initially HER2-positive tumor, which confirmed HER2 status at relapse (Fig. 2a,b). Any attempts to generalize evidence from two cases would miserably fail. We tried to add to this relevant issue by analyzing data on the change/stability of HER2 status by variables related to the metastatic spread. The only relevant result we observed was the significantly less common occurrence of brain metastasis in women with a change in HER2 status (p: 0.01), while some evidence emerged of more frequent visceral involvement in this same group (p: 0.054). In addition, in future studies with prospective design, we foresee the inclusion of investigational tasks related to the use of circulating tumour cells (CTCs), whose dynamics in peripheral blood may recapitulate quali/quantitative aspects of HER2 expression at the tissue level^26–29^.

In brief, our results encourage considering the hypothesis that HER2-discordant patients should be distinctively considered with respect to HER2-concordant patients. Indeed, our findings elicited some evidence of possibly relevant differences from both a clinical-biological and from a prognostic standpoint, although such evidence was not fully statistically significant. However, the previously mentioned sources of bias invite caution in our results’ interpretation and generalization. Additional, adequately sized, ad hoc trials are warranted in molecularly defined subgroups to define the most appropriate therapeutic approaches in light of HER2-status discrepancies. This attempt hold particular relevance in the presence of the new anti-HER2 targeted agents, which have only recently widen the prior therapeutic scenario and whose efficacy could not be taken into account in prior studies. In the subset of HER2-discordant patients expressing one or both hormonal receptors, hormonal manipulations together with anti-HER2 treatments could become the mainstay of treatment. In these tumors, treatment de-escalation may represent a valid option, since the benefit coming from chemotherapy still remains undefined.

## Materials and methods

Our study cohort included 491 patients with HER2-positive mBC treated at 45 Italian cancer centers with pertuzumab-based-therapy and/or T-DM1. The observational study had a retrospective approach: key demographic, anthropometric and clinical-pathological data were collected through medical records, together with information concerning treatments administered and clinical outcomes.

Patients were suitable for inclusion if aged 18 years or older, initially diagnosed with an early BC, irrespectively of its HER2 status, and subsequently with HER2-positive mBC, defined as a BC spreading beyond the mammary gland and the pertinent locoregional lymph nodes, including the supraclavicular ones. Each patient contributing data to the present analysis had received at least one cycle of a pertuzumab and/or T-DM1 regimen according to current guidelines.

Clinical outcomes were evaluated by RECIST criteria. Data were anonymized and entered into a dedicated database with a SPSS operating interface. Pathology assessment was performed in surgical specimens of primary tumors and, whenever available, in bioptic samples of metastatic lesions by expert pathologists at the participating centers, as per national standards. Estrogen (ER) and progesterone (PgR) receptors status were determined at each center by IHC assays according to the local standards. Positivity was considered at a cutoff of ≥ 1%. HER2 assessment was performed based on the 2013 ASCO-CAP guidelines and their 2018 update. A positive HER2 status required an IHC score of 3 + or positive fluorescence/chromogenic/silver in situ hybridization (FISH/CISH/SISH)^12,13^.

The study was coordinated by the Regina Elena National Cancer Institute, upon approval of the institutional ethical committee of the coordinating and satellite centers. The study was carried out in compliance with the Helsinki Declaration. A written informed consent was obtained by all patients that contributed data to this analysis.

### Statistical analysis

The variables of interest were studied using descriptive analyses. Categorical variables were addressed by χ2 or Fisher exact test. Continuous variables were reported in terms of means/medians and standard deviation/ranges. Endpoints for efficacy outcome included progression‐free survival (PFS) and overall survival (OS). Progression free survival following first line treatment was calculated from the time of treatment start to the disease progression, interruption for toxicity, death or lost to follow‐up. Survival analysis was performed by the Kaplan–Meier product-limit method from the date of clinically-instrumentally assessed metastatic spread until disease progression or death. The log-rank test was used to elicit differences between subgroups. Significance was set at a *p* ≤ 0.05 level. Hazard Ratio (HR) and the 95% confidence intervals (95% CI) were estimated using the Cox univariate model. A multivariate Cox proportional hazard model, in order to compare the prognostic impact of several factors on OS, was developed using stepwise regression (forward selection). Enter limit and remove limit were *p* = 0.10 and *p* = 0.15, respectively. All statistical computations were performed using the SPSS software (SPSS version 21.0, SPSS Inc., Chicago, IL).

## Supplementary Information

Supplementary Information 1.

Supplementary Information 2.

Supplementary Information 3.

## Data Availability

The datasets generated during and/or analysed during the current study are available from the corresponding author on reasonable request.
