# Supplementary material for: The prognostic relevance of HER2-positivity gain in metastatic breast cancer in the ChangeHER trial
Source: Sci Rep. 2021 Jul 2;11:13770. doi: 10.1038/s41598-021-92774-z (PMC8253801; doi:10.1038/s41598-021-92774-z)
Supplement: Supplementary file 3 — Supplementary Information 3. [file 41598_2021_92774_MOESM3_ESM.doc]

**TITLE PAGE**

**The prognostic relevance of HER2-positivity gain in metastatic breast cancer in the ChangeHER trial**

Laura Pizzuti, Maddalena Barba, Marco Mazzotta*, Eriseld Krasniqi, Marcello Maugeri-Saccà, Teresa Gamucci, Rossana Berardi, Lorenzo Livi, Corrado Ficorella, Clara Natoli, Enrico Cortesi, Daniele Generali, Nicla La Verde, Alessandra Cassano, Emilio Bria, Luca Moscetti, Andrea Michelotti, Vincenzo Adamo, Claudio Zamagni, Giuseppe Tonini, Domenico Sergi, Daniele Marinelli, Giancarlo Paoletti, Silverio Tomao, Andrea Botticelli, Paolo Marchetti, Nicola Tinari, Antonino Grassadonia, Maria Rosaria Valerio, Rosanna Mirabelli, Maria Agnese Fabbri, Nicola D’Ostilio, Enzo Veltri, Domenico Corsi, Ornella Garrone, Ida Paris, Giuseppina Sarobba, Icro Meattini, Mirco Pistelli, Francesco Giotta, Vito Lorusso, Carlo Garufi, Antonio Russo, Marina Cazzaniga, Pietro Del Medico, Mario Roselli, Angela Vaccaro, Letizia Perracchio, Anna di Benedetto, Theodora Daralioti, Isabella Sperduti, Ruggero De Maria, Angelo Di Leo, Giuseppe Sanguineti, Gennaro Ciliberto, Patrizia Vici.

**Supplementary Table 2.** Treatment administered as second line in the study participants based on HER2 status at initial diagnosis of breast cancer. Abbreviations. N: Number; CT: chemotherapy; ET: endocrine therapy.

| **Treatments** | **HER2-negative, 102 pts**  **[N (%)]** | **HER2-positive, 389 pts**  **[N (%)]** |
| --- | --- | --- |
| T-DM1 | 66 (64.7) | 198 (50.9) |
| Trastuzumab + CT | 12 (11.8) | 62 (15.9) |
| CT alone | 2 (2.0) | 12 (3.1) |
| ET alone | - | 3 (0.8) |
| Lapatinib + Capecitabine | 4 (3.9) | 15 (3.8) |
| Unknown | 1 (0.9) | 14 (3.6) |
| No therapy | 17 (16.7) | 85 (21.9) |
